# Measuring reading behavior in large-scale assessments and surveys

**DOI:** 10.3389/fpsyg.2022.1044290

**Published:** 2023-02-02

**Authors:** Franziska Maria Locher, Maik Philipp

**Affiliations:** ^1^Department of Educational Assessment, St.Gallen University of Teacher Education, St. Gallen, Switzerland; ^2^Department for Higher and Adult Education, Zurich University of Teacher Education, Zurich, Switzerland

**Keywords:** reading behavior, print exposure, reading activity, measurement, large-scale assessment

## Abstract

Large amounts of studies have shown that reading behavior is an important aspect for the development of students’ reading skills. The construct reading behavior as examined in large-scale assessments and surveys within the field of empirical educational research is operationalized through a wide range of reading and reading-related aspects (e.g., reading time, reading frequency, print exposure, reading engagement, book genre preferences, knowledge of authors or book titles) and a broad array of measurement methods are used. The approaches to measure the same variable – namely reading behavior – differ fundamentally from each other, while at the same time, a clear concept that would help to classify the used measurement instruments and to interpret them in relation to the superordinate construct of reading behavior is missing. Therefore, the present article aims to give an overview of methods to measure reading behavior within the context of large-scale assessments and surveys, and to discuss how they were implemented. Finally, we make some suggestions on how it might be possible to relate the applied measurement approaches to each other, especially in terms of their content and theoretical relationship to the overarching construct of reading behavior.

## 1. Introduction

As reading skills are one of the basic qualifications required for participation in cultural, political, and economic life (OECD, 2003; Yamashita et al., 2018), many studies have investigated variables that relate to the development of such skills. A person’s reading behavior has shown to be one of the most important predictors of reading skill development: A great number of studies provide convincing evidence on the positive relation between reading skills and reading behavior in childhood as well as in adolescence and beyond. Already in preschool years, parent–child book reading activities have shown to be related to young children’s oral language and reading skills (e.g., Bus et al., 1995; Burgess et al., 2002; de Bondt et al., 2020; for an overview). Furthermore, research on primary and secondary school students has revealed that reading amount and measures of time spent reading significantly predict later reading skills, such as text comprehension (e.g., Anderson et al., 1988; Pfost et al., 2013; Jerrim et al., 2020; Locher and Pfost, 2020). In addition, further studies analyzed preferences for specific reading content such as reading newspapers and magazines or analyzed emotions towards reading and books in relation to different facets of students’ reading skills, and again, at least in parts, found such positive relations (Stanovich et al., 1995; Spear-Swerling et al., 2010; Kirby et al., 2011). The importance of reading is further supported by studies reporting a positive relation between the recognition of authors (author recognition test; ART) or book titles (title recognition test; TRT) and later reading skills, meaning that students who recognized a high number of book titles or author names exhibited higher reading comprehension, decoding and spelling skills (e.g., Mol and Bus, 2011; for an overview).

All studies cited above refer to variables that are assumed to reflect a person’s reading behavior. However, they cover a wide range of different reading and reading-related aspects that often differ fundamentally from each other. The aim of the present article is to give an overview of popular methods that have come into use to measure reading behavior, especially within the context of large-scale assessments and surveys in primary and secondary education, and to discuss how they are implemented. The focus of the article is not on promoting theoretical work regarding the definition of the reading behavior construct. Nevertheless, we want to end up with a proposal on how to compare different applied measurement approaches. This takes the content and theoretical relationship to the overarching construct of reading behavior into account.

## 2. Reading behavior – A provisional definition as a basis for the discussion of the methods measuring reading behavior

Researchers in the field of education have not yet found a consensus with regard to the term reading behavior. Therefore, as a starting point within this article, we want to provide a provisional definition of the construct of reading behavior, as a common basis for understanding.

The starting point of our provisional definition of the reading behavior construct is the idea of a four language system (Berninger, 2000), which differentiates between listening, speaking, reading, and writing according to modality (oral language vs. written text) and an input or output dimension. According to this idea, reading is the process of gaining input from written text. Second, rather than merely regarding reading as a passive, receptive process, our definition should incorporate the idea that reading is an active process whose purpose is to extract and construct meaning (Kintsch and Rawson, 2005; Perfetti et al., 2005; Snow, 2010). Given these premises, in the context of the subsequent discussion, we define the reading behavior construct in relation to the reading activity. Reading activity is an interaction between a person (the reader) and written text (print) whose goal is the construction of meaning. Although the act of reading typically involves complex cognitive processes, such as semantic integration or meta-cognition (e.g., Kintsch and Rawson, 2005; Schindler and Richter, 2018), these processes are not explicitly addressed within the current paper. Our provisional definition includes a broad range of activities, regardless of what is read, when, where or why a text is read, or how long the text is. Therefore, four criteria (1) Reading activity and reading-related activity, (2) Reading activities in a specific situation and general tendencies, (3) Quantity and quality of reading activities, and (4) Leisure and school reading activity for a further clarification and specification of the nature of the superordinate construct reading behavior will be applied within the paper. These are described next.

### 2.1. Reading activity and reading-related activity

Reading behavior as we define it includes a broad range of activities that are related to the interaction between reader and written text. The central facet of the reading behavior construct is, of course, reading itself, in the sense of an activity whose purpose is to extract and construct meaning from a specific text (see definition above; Kintsch and Rawson, 2005; Perfetti et al., 2005; Snow, 2010). Beyond the reading activity itself, however, activities that are distally related to reading, such as library visits, buying books, or communication about texts, can also be considered a facet of the reading behavior construct, as there is also interaction between reader and text. The different activities should not be considered equivalent.

### 2.2. Reading activities in a specific situation and general tendencies

The interaction between the text and the reader can further refer to a specific situation (e.g., Has a person been engaged in reading on the weekend?) or to larger periods of time in which activities are considered cumulatively (e.g., How often has a person typically been engaged in reading on weekends in the last 6 months?). The latter is what may be considered a reading habit. Following Schmidt and Retelsdorf (2016), interactions or activities have “to have been executed successfully and repeatedly in the past to form a habit” (Schmidt and Retelsdorf, 2016, p. 2). However, they remark that in large-scale assessments and surveys, even when studies examining reading seem to refer more to a specific situation (e.g., reading volume measured *via* reading diary), the concept of habit often forms the theoretical foundation. In the area of reading motivation, there are attempts to distinguish between situative and habitual reading motivation and there are studies showing that individual situations can have an effect on habitual motivation (e.g., Guthrie et al., 2005, 2007; Locher et al., 2019). Accordingly, it seems to be worth it to make a clear distinction between reading activities in a specific situation and general tendencies – both in measurement and its theoretical foundation.

### 2.3. Quantity and quality of reading activities

Moving beyond the simple assumption that reading activities vary with respect to quantitative aspects of reading, such as the time spent reading, this idea can be extended to qualitative differences in reading. This extension follows the assumption that reading activities can be distinguished from each other by the reading material and thus might differ in their correlates with other variables. For example, it has been found that reading fiction in particular is closely related to the development of reading skills, whereas only weak, in some cases even negative correlations are found for reading newspapers and magazines or reading on the internet – especially reading social media (e.g., OECD, 2010; Pfost et al., 2013; Jerrim and Moss, 2019; Torppa et al., 2019). However, qualitative aspects of reading behavior are multifaceted, including many different aspects in terms of the nature of the reading material, such as text difficulty and readability (e.g., Fry, 1968; Klare, 2000; Norris and Ortega, 2009) or text content, which may also relate to differences in the individual mode of reading (e.g., deep reading vs. shallow reading; see also Delgado et al., 2018, for discussion). A further differentiation that tends to be important to consider is whether a text is read online, respectively, on digital devices or analog, meaning as a paper-printed text. We think this differentiation is important for two reasons. On the one hand, text that is read online often itself differs fundamentally from text typically read on paper-based media with regard to characteristics such as the integration of multimedia features or the availability of source information (Magliano et al., 2018). On the other hand, readers themselves tend to interact differentially even when confronted with equal text, probably due to differences in the processing style with regard to the written information or cognitive load (e.g., Delgado et al., 2018).

### 2.4. Leisure and school reading activity

Reading always takes place in contexts (e.g., Rouet et al., 2017). Therefore, the context of a reading activity should be taken into account. In empirical educational research, the most common approach to consider the context is to compare a person’s reading behavior at school with the person’s reading behavior in their leisure time (e.g., Guthrie et al., 1999; Liew et al., 2020). Reading in and for school is assumed to be predominantly externally regulated (e.g., by the teacher, tasks, curriculum). In leisure time, by contrast, the choice of text is more internally regulated, which means that people decide for themselves or more autonomously which texts they want to read (e.g., Ivey and Broaddus, 2001; De Naeghel et al., 2012; McKenna et al., 2012). Therefore, students’ reading behavior might be different in the two broader contexts.

As mentioned before, the definitional discussion of the construct of reading behavior in this section is to be understood merely as a provisional working definition. The purpose of this provisional working definition is to be able to discuss and evaluate the methods or approaches used to measure reading behavior comprehensively, i.e., also in terms of content. This discussion and evaluation will follow in the next section.

## 3. Methods that aim to measure reading behavior within large-scale assessments and surveys in primary and secondary education

In this section, we want to give an overview of three methods to measure reading behavior that are commonly used in large-scale assessments and surveys in primary and secondary education: questionnaires (global evaluation, differentiated evaluation, and item sets), reading diaries and checklist procedures. Beyond a rather general discussion of these methods, referring to relevant literature dealing with advantages and disadvantages using them (e.g., Fahrenberg et al., 2007; Gogol et al., 2014; Allen et al., 2022; Wimmer and Ferguson, 2022), we would like to go into more detail. Therefore, we will share reflections on the specific measurement approaches from example studies in the area of large-scale assessments and surveys, which aim to examine the relation between reading behavior and reading skills in primary and secondary school students (see Table 1 for a summary). The following section is an illustrative selection of studies representing as broad as possible a range of instruments to measure reading behavior. Since we do not aim at giving an exhaustive overview of all measures that have been employed we chose prototypical approaches stemming from the empirical research. Studies were considered relevant for the present paper after an intensive discussion among the authors, paying attention to various aspects such as sample characteristics (we only included studies with students in primary and secondary education as the target group), publication type (we only included studies from peer-reviewed journals, to ensure a minimum of a quality standard), content (reading behavior should be at least one focus of the study and not just one control variable among many), and method (broad as possible range of methods and/or instruments).

**Table 1:** Overview of advantages and disadvantages of the three methods to measure reading behavior in large-scale assessments and surveys in primary and secondary education.

**Method**		**Strengths**	**Limitations**	**Conclusion**
**Questionnaire**				
	**Global Evaluation** e.g.: About how much time do you usually spend reading?	Efficient and time saving Easy to implement	Scale contains no information about qualitative aspects of reading Coarse-grained estimator which only records relative differences between students Bias due to memory effects or social desirability	*Optimal method for large studies with time restrictions and studies focusing on quantitative aspects of reading.* But… Clear specification of reading context needed (leisure reading is not equivalent to reading for enjoyment) Decide whether asking about reading frequency or time is more meaningful Define what reading material should be considered
	**Differentiated Evaluation** e.g.: How often do you usually read (1) magazines/ newspapers, (2) fiction, (3) nonfiction?	Relatively efficient and time saving Allows considering and analyzing different reading materials	Distinguishing different reading activities may be cognitively demanding, especially for younger children (differentiation/classification of text types) Bias due to memory effects or social desirability	*Optimal method for studies focusing on quantitative aspects of reading in which information regarding different text types is needed.* But… Clear specification of reading context needed (leisure reading is not equivalent to reading because one wants to) Carefully think about the response scale (frequency or time) and text categories (e.g., consider participant age)
	**Item Sets** e.g., combining a variety of items	Methodological advantages (e.g., latent variable estimation)	Sometimes hard to say what is really measured /operationalized (e.g., conceptual proximity of included items to reading motivation) Interpretation of scores might be unclear when diverse items are included Bias due to memory effects or social desirability	*Optimal method when a study wants to cover a broader definition of reading behavior.* But… Be careful when analyzing and interpreting item sets (correlation between reading motivation and a reading behavior construct including motivational aspects might be higher than with constructs focusing solely on quantitative aspects of reading) Best to exclude items related to reading motivation or attitudes towards reading
**Reading Diary**		Continuous documentation of reading behavior Precise estimation Possible to get data on absolute reading times Allows deep insights	Very complex and time intensive High strain on test persons Often data collection is only possible for short periods of time and therefore might be difficult to generalize Measure might also act as an intervention	*Optimal method to measure reading behavior in detail (e.g., concrete reading material).* But… Data quality strongly depends on the study design (e.g., long enough time period, event-based design potentially more precise than end-of-day diaries) Consider whether method is really needed (e.g., when only reading time is of interest, global evaluation might require fewer resources)
**Checklist Procedures** e.g., ART & TRT		Tends to avoid socially desirable response behavior Does not require retrospective time judgements	Indirect measure of reading behavior Time- and space-consuming in survey design Instrument strongly dependents on item selection Scales are difficult to compare across studies Remains unclear whether the book was actually read Movie adaptations of books might falsify results	*Method especially has potential as a proxy for reading behavior in very young children that goes beyond the home literacy environment.* But… Be careful when interpreting scores (recognition tests as a black box), especially in adolescence and young adulthood Checklists should be used in addition to other reading behavior measures

### 3.1. Reading behavior measured by using questionnaire data

#### 3.1.1. Global evaluation and differentiated evaluation

In empirical educational research, a large proportion of studies use global retrospective self-report data from questionnaires (e.g., Fahrenberg et al., 2007). One popular measures is a global retrospective self-report measure of reading behavior by one item. Within this one item, participants might be asked to indicate the time spent reading per day, as found, for example within PISA 2018 (OECD, 2018) or studies such as by Miyamoto et al. (2019) based on data from the German National Educational Panel Study (NEPS). Alternatively, participants might be asked to indicate reading frequency in sense of how often they read, as for instance in Pfost et al. (2010). Both options exist in the literature and the selection of a response scale is meaningful. Reading frequency tends to be a more coarse-grained indicator. Asking about reading time, on the other hand, requires quite precise reporting and, above all, reflection by the respondent, e.g., estimating the mean reading time across many days. Therefore, asking how often participants read might be a more suitable indicator in cases in which respondents tend to be less aware of their reading behavior.

Global evaluations aim to summarize reading time or frequency for a large variety of reading materials, from books to e-mails and magazines. However, some studies using this approach, did not specify what kind of reading should be included when indicating the time spent reading per day (e.g., Pfost et al., 2010). This might be problematic, as respondents might vary unintentionally in their response behavior due to this lack of information (e.g., some exclude and some include reading blogs or e-mails). Therefore, it is advisable to explain what kind of reading and what reading material should be considered, as is the case in PISA 2018 (OECD, 2018)^1^ or the paper by Miyamoto et al. (2019)^2^. Directly referring to quantitative aspects of reading, such single-item measures are often used within large-scale assessments and surveys due to their economic and easy implementation. Although single-item measures and short scales are often criticized for psychometric disadvantages (e.g., low reliability; see Allen et al., 2022, for a critical discussion), their use may be nevertheless recommended in studies with strong time restrictions and when the construct is concrete, unidimensional in content and reflecting the subjective experience (Gogol et al., 2014). However, the information provided by such a global evaluation of reading time or frequency is limited to the average trend.

To address this limitation, measures such as a text-specific evaluation of reading time and frequency are often additionally used (e.g.: *How often do you usually read (1) magazines/newspapers, (2) fiction, (3) nonfiction?*). Between studies, the categorizations of text types or genres can vary recognizably. While some studies include only three or four quite global categories (e.g., Cantrell et al., 2018), other measures ask about 14 or more text types, which were then subsequently categorized *via* exploratory factor analysis (e.g., Torppa et al., 2019). Often, studies also differentiate in some way between “digital” texts and other categories, such as book reading or newspaper reading (e.g., Cantrell et al., 2018; Torppa et al., 2019). Within these studies, “digital” texts were typically defined as blogs, factual websites or e-mails. Therefore, such measures consider reading on the Internet and not digital reading in sense of reading on digital devices (e.g., reading an online newspaper). Thus, while it is commendable that the studies considered reading on the Internet (labeled “digital texts”) as a separate category, this approach fails to consider that the other categories, such as books or magazines, can also be read on digital devices, making them digital texts. Using this categorization, mode of presentation and text content are intertwined.

Although text specific measures may add important information about how often or how long individuals spend time on average reading different reading materials (e.g., Pfost et al., 2013; Jerrim and Moss, 2019), distinguishing between different reading activities may also be a challenge. For instance, responding to such scales may be quite cognitively demanding, especially for younger students. Locher and Pfost (2019), for example, have shown that even fifth graders still had severe difficulties differentiating between fine-grained categories of reading material. It is not always the case that participants are asked for general reading behavior. For instance, McGeown et al. (2015) asked students to report their reading time for a specific period, namely during the previous weekend; they were not asked to estimate their average reading behavior in general. On the one hand, it might be easier for students to answer more precisely about their reading behavior over a specific period (smaller memory effects). On the other hand, reading activities within quite limited periods of time might not represent students` general reading behavior or so called reading habits (generalizability).

The global and differentiated evaluation of reading time or frequency might refer to reading done in all sorts of situations and contexts. For example, some studies ask about reading for enjoyment, as in the global evaluation from PISA 2018 (OECD, 2018) and Pfost et al. (2010). Others such as Miyamoto et al. (2019) ask about reading outside school, and still others such as McGeown et al. (2015) or Torppa et al. (2019) explicitly focus on reading done during leisure time (e.g., the weekend). In the differentiated evaluation within PISA 2018 (OECD, 2018), students were asked about self-initiated reading conducted voluntarily (reading “because you want to”). This decision is meaningful. For instance, the version chosen by PISA 2018 (OECD, 2018) has consequences that might be problematic for some research questions, as reading for enjoyment *via* definition overlaps with intrinsic motivation. Alternatively, the version selected by Miyamoto et al. (2019) based on NEPS data might not be specific enough, as it can also include reading school-related texts and books outside school.

#### 3.1.2. Item sets

Alongside global and text type specific evaluations of reading time, it is also possible to use item sets. These reading scales combine a variety of items (e.g., daily reading time, library visits, the wish to receive books as gifts or an enjoyment of leisure reading) and are often used to form either a manifest or latent reading behavior construct. Item sets provide wide degrees of freedom to design and define a construct. However, there is a lack of validated item sets (Wimmer and Ferguson, 2022). In consequence, different studies use different approaches, at least to a certain extent. For instance, Troyer et al. (2019) refer solely to the reading activity itself and quantitative aspects of reading in their scale, which was adapted from Schaffner et al. (2013). Guthrie et al. (1999) also used a measure of reading activity in which they (alongside quantitative aspects) ask students to name a book’s title or main topic or the author of a book in a specific genre they read. However, this information was not used to gather further information about the text (e.g., text difficulty, content). Stanovich et al. (1995) and McElvany et al. (2008) include items referring to the reading activity itself as well as rather distal aspects (e.g., wish for books). Interestingly, Stanovich et al. (1995) included an item asking about reading motivation or reading enjoyment. It is even more a challenge to interpret the construct measured in this case, as the scale contains a mixture of different aspects related to reading. In a relatively recent study by Schmidt and Retelsdorf (2016), the authors’ adapted Reading Habit Scale tends to focus more on reading-related attitudes rather than information about reading activities. This is particularly evident from the fact that the introductory sentence for the 10 items asks how students feel about recreational reading. Consequently, the item set procedure can result in conceptual overlap between reading behavior and other constructs, such as reading motivation (Schroeder et al., 2016).

As within global and differentiated evaluations, also within item sets the reading context plays a crucial role. For example, Guthrie et al. (1999) explicitly examined differences in reading context. In their study, they aimed to measure children’s amount of reading with respect to different genres and two contexts: reading for school (e.g., science) and reading for personal enjoyment (e.g., romance). However, romance books might also be read in school, while science books can be read during leisure time. Troyer et al. (2019) and Schmidt and Retelsdorf (2016) provide some information on the reading context, as they ask about reading during the summer school holidays or during leisure time. In contrast, the context remains rather nonspecific in most studies.

Although retrospective self-reports (e.g., global evaluation, differentiated evaluation, and item sets) tend to be quite popular measures of (reading) behavior, some researchers (e.g., Fahrenberg et al., 2007) strongly question whether such measures really capture persons’ behavior, as there are several possibilities for bias. For instance, retrospective self-report data can be affected by cognitive schemata, social stereotypes, response tendencies or memory effects (Fahrenberg et al., 2007). In addition, self-reports in questionnaires are likely to suffer from social desirability bias (Mol and Bus, 2011).

### 3.2. Reading behavior measured by using reading diaries

Another possibility to gather information is to use reading diaries, in which people document their reading activities, meaning how long and which books, magazines, newspapers or other texts they read (e.g., Anderson et al., 1988; Nieuwenboom, 2008; Chen and Fang, 2015; Stoffelsma, 2018). Reading diaries are often seen as a kind of gold standard, as they promise to quite precisely document students’ reading activities and provide concrete information about books or texts that have been read that can be used for further analyses. Again, however, data quality depends on the study design. Anderson et al. (1988), as an example, asked students to document their activities over 26 weeks, which is a quite long time for a diary study and speaks in favor of the quality of the data in the sense of generalizability. In comparison, Greaney and Hegarty (1987) only had students to document their activities for a few days. Their study used daily diaries, which were divided into half-hourly sections, and pupils could record their leisure activities on each of four predetermined days. These were a Sunday, a Tuesday and a Thursday during 1 week, and a Monday on the following week. Whereas longer time periods might result in better representativeness of the diary data for the habitual behavior, longer time periods might also go along with more missing data, measurement errors, and unwillingness to participate in the study and boredom effects (Bolger et al., 2003). Diaries are mostly filled in retrospectively once a day (e.g., end-of-day diary or on the next day at school). But filling out the diary after too much time has passed increases the risk of a retrospective bias when people have to estimate their activities (Bolger et al., 2003; Wilhelm et al., 2012), for instance as students might forget about specific activities or the duration of their reading activities from the preceding day.

Most daily reading diary studies have relied on the paper-pencil method (Bolger et al., 2003; Fahrenberg et al., 2007; Wilhelm et al., 2012). However, this method is quite complex and time consuming, meaning that it can be a substantial burden for participants (Bolger et al., 2003; Fahrenberg et al., 2007). For instance, participants have to carry their diaries with them at all times and make entries every day over a sufficiently long time period, a constraint that tends to restrict its use in practical research. Taking into account that reading diaries are associated with high costs and effort, it needs to be discussed in what situations reading diaries are necessary and appropriate. For instance, although participants in the studies by Greaney and Hegarty (1987) and Anderson et al. (1988) documented their leisure activities in an extensive and elaborate way as well as provided responses about reading time with respect to different genres or text types, the studies’ authors only made use of average documented reading time to explore the relation with reading skills. Thus, various qualitative aspects of reading were measured, but not used for the subsequent analyses.

In addition to paper-pencil diaries, an electronic documentation of reading behavior – that is, an ambulatory assessment – might come into use (e.g., Locher et al., 2022). Ambulatory assessment “refers to the use of computer-assigned methodology [e.g., smartphones] for self-reports, behavior records, or physiological measurements, while the participant undergoes normal daily activities” (Fahrenberg et al., 2007, p. 206). There are numerous advantages to using an ambulatory assessment to assess reading behavior compared to paper-pencil diaries, e.g., data can be collected in daily life at low cost, participants can fill out the reading diary easily whenever and wherever they want, a better control of the timing and reliability of the entries, and finally a higher compliance (Bolger et al., 2003; Fahrenberg et al., 2007).

### 3.3. Reading behavior measured by using checklist procedures (title, magazine, and author recognition tests)

The third method that is often used in large-scale assessments and surveys in primary and secondary education are checklists, which are variously known as title, magazine and author recognition tests (TRT, MRT, & ART; Stanovich and West, 1989). In these tests, students have to indicate whether they recognize a book, a magazine title or an author’s name from a list containing existing and non-existing titles, respectively, authors. Checklist procedures rely on the fundamental idea that while reading, people learn about book titles or authors they have read. In a cumulative sense, the more a person has read in her lifetime, the more book titles and authors the person should be able to recognize (Wimmer and Ferguson, 2022). In other words, these measures aggregate knowledge acquired over many situations which is correlated with actual reading activities. Alongside adolescents and adults, recognition tests are also popular to use in studies of preschool children (Grolig et al., 2017). For example, in the ART developed and validated by Stanovich and West (1989), 50 author names and 50 foils were included. The 50 authentic authors were “popular” authors. “Highbrow” authors (= only known by highly educated readers) were not considered. Beyond that, the researchers aimed to include writers from a wide variety of genres (nonfiction, such as sports, humor, and history; fiction, such as mystery, romance, Westerns, science fiction). In addition to the ART, a MRT was also developed and validated. Similarly to the ART, the MRT comprised the names of 50 popular magazines and 50 foils. The magazines came from a mix of contents (e.g., sports, gossip, technology). Unlike Stanovich and West (1989), Goff et al. (2005) used a TRT comprising just 20 authentic children’s book titles and 20 foils. As in Stanovich and West (1989), the titles covered a wide range of types of literature, but limited to reading ages within primary schools.

For Stanovich and West (1989), one important criterion was to include only authors who are not regularly studied in the school curriculum, as they aimed to measure out-of-school/free-time reading volume. Goff et al. (2005) included no such criterion or specification with respect to the reading context. While Stanovich and West’s (1989) approach – only including authors that are not part of the curriculum – represents a good method of limiting the reading context considered, it can nevertheless be questioned whether exclusively extra-curricular reading was measured. On the one hand, literary class may encompass more than reading authors from a fixed curriculum. On the other hand, there are many ways to learn about book titles or author names other than reading the book – especially in adolescence and early adulthood. For instance, if there is a movie based on a book, persons might recognize an author or book name because they have watched or heard about the movie. People might also read titles or author names on bestseller lists, weblogs or in newspapers. Therefore, checklists especially when used in older students or adults might measure further facets other than just reading behavior. Another way to use checklist procedures is Tremblay et al.’s (2020) retrospective recognition test, in which they aimed to measure adolescents’ storybook reading when participants were children. The checklist included 25 popular children’s titles and 8 foils. However, it has to be considered that participants might check titles that they currently recognize (e.g., because they are regularly engaged in joint reading activities with younger children), but which these persons probably would not have recognized when they were younger themselves.

In general, recognition tests are very popular for studies of all age groups because this indirect method is a good way to deal with socially desirable response behavior. However, the fact that ARTs, MRTs, and TRTs are indirect methods can also be a limitation, as recognizing a book does not automatically mean that he or she has read it (Schroeder et al., 2016). This differentiation in author and title knowledge is further supported by a study of Martin-Chang and Gould (2008), who found that knowing authors through secondary sources such as conversation and knowing authors through personal reading were not correlated with each other. In addition, knowing authors by personal reading was related to further measures of reading behavior and, in comparison to the knowledge of authors by secondary sources, was more strongly related to different reading skill measures. Furthermore, recognition tests may not prove informative for non-native speakers (McCarron and Kuperman, 2021). Consequently, the simple use of author, title or magazine checklists might not in all cases be a satisfying indicator of a students’ reading behavior. Therefore, checklist procedures are often used in combination with other methods, such as item sets (e.g., Stanovich et al., 1995) or reading diaries (e.g., Allen et al., 1992). This is further supported by a recent study of Wimmer and Ferguson (2022) who showed that although construct validity of both self-report measures and ART were on a good level, the two methods may not be used interchangeably.

## 4. Interim conclusion

In the context of this article, we defined reading behavior provisionally as a broad construct that encompasses various activities related to the interaction between a person (the reader) and written text (print). We defined four criteria (1) Reading activity and reading-related activity, (2) Reading activities in a specific situation and general tendencies, (3) Quantity and quality of reading activities, and (4) Leisure and school reading activity to further clarify the character of the superordinate reading behavior construct, as reading (related) activities differ fundamentally from each other. Having in mind these four criteria when discussing the three methods, it became apparent that there is substantial variance in the applied measures in terms of how the methods are implemented. Item sets that only refer to the reading activity itself cannot be compared with item sets that also include motivational aspects. Similarly, global evaluations of *reading time* in a *leisure context* cannot be compared with global evaluations of *reading frequency* in a *school context*. Comparisons are further impeded if it is not specified which reading material was considered by the students when responding to the questions (e.g., including digital reading or not).

The finding that reading behavior is operationalized in quite different ways is *per se* not a reason for concern. However, the applied measures differ significantly in their nature and in their relation to the overall reading behavior construct, while a clear theory or concept explaining this variation tends to be missing at the same time. For instance, McGeown et al. (2015) labeled their construct “reading habits” and used a question that asked about time spent reading in a specific time period, namely on weekends. Schmidt and Retelsdorf (2016) or Stanovich et al. (1995) also examined a construct labeled reading habits, however, their item sets include aspects of reading motivation or attitudes towards reading. We recognized that similar labels are used for different reading facets (situation specific reading time vs. habitualized attitudes towards reading). In addition, studies often used apparently similar methods but when taking a closer look, these studies differ in small but important details (e.g., What reading material is included? How is the reading context defined?) – small details that might lead to differences in the participant responses and therefore change the findings.

Table 1 gives an overview on strengths and limitations of the three methods and includes a summary of the discussed reflections on the specific measurement approaches. But the variance mentioned above hinders the derivation of a general recommendation for one of the three methods. Therefore, on the basis of the four criteria to clarify the reading behavior construct and based on the findings from the discussion of the example studies, we want to present a possibility for evaluating similarities and differences in the applied measures between different studies and its approaches to measure reading behavior used within large-scale assessments and surveys. With this proposal for a multidimensional distance measurement, it should be possible to compare and evaluate the used measurement approaches.

## 5. A proposal for a multidimensional distance measurement

Table 2 shows four dimensions (see also section 2 for details) including different categories for a differentiation on which the applied measures may be evaluated. We use two of the discussed example studies from section 3 to illustrate how large the differences in the item set method can be and might be made visible. In the following, we will explain the proposal for a multidimensional distance measurement in detail.

**Table 2:** Multidimensional classification system for reading behavior measures in large scale assessments and surveys.

Dimension	Categories for a systematic differentiation	Example (method item set)	
		Stanovich et al. (1995)	Troyer et al. (2019)
Proximal-to-Distal	Reading activity itself (proximal)	Reading frequency, number of books	Number of books, reading time, reading frequency
	Preceding and succeeding reading activities	Purchase of magazines, reading enjoyment	–
	Indirect relations to reading activities (distal)	–	–
Specific-to-General	Reading activity in a specific situation/time period	–	During summer
	General reading tendencies	Reading in general	–
Quantity-to-Quality	Global (explicitly stated)	–	Book reading
	Global (not explicitly stated)	–	–
	Different reading material specified	Magazines, newspapers, books	–
School-to-Leisure	School reading	Not stated	Not stated
	Leisure reading	Not stated	Spare time during summer

### 5.1. Proximal to distal

First, there are measures that refer to direct reading activities, such as the time spent reading, the frequency of being engaged in reading or qualitative aspects of the activity of reading (= proximal reading facet). Second, there are measures that refer to or include aspects that are more distal but still closely related to the activity of reading, as they often (1) precede specific reading activities or (2) occur subsequently to reading. Therefore, reading-related activities within this category imply that (1) a person/a student is probably going to become engaged in reading (e.g., buying a book for future reading) or that (2) something has been read by the person (e.g., communication with friends or family about books and other texts that have been read). Both facets indicate further engagement with the reading material. Third, there are measures that refer to aspects that are only indirectly related to actual reading activities. For example, print exposure measures based on the recognition of different book titles or authors’ names (see Allen et al., 1992; Mol and Bus, 2011) might be assigned to this category, as these measures refer to knowledge acquired by regular engagement in reading activities.

### 5.2. Specific to general

With respect to this dimension, we can differentiate between two measure categories: either participants are asked about reading activities in a specific situation or a limited time period, or alternatively, participants are asked about reading activities in general. With respect to this dimension, we want to point out that each operationalization does not necessarily correspond with the theoretical assumptions behind it. McGeown et al. (2015), for instance, aimed to measure “reading habits” by asking about time spent reading on the last weekend. Already Schmidt and Retelsdorf (2016) raised awareness to this problem that even when studies examining reading that seem to refer more to a specific situation, the concept of habit often forms the theoretical foundation.

### 5.3. Quantity to quality

Moving beyond the question of how often persons read or how much time they spend reading, the applied measures can be classified into whether a study aimed to gain differentiated information on the reading material (mostly text genre and medium; e.g. Pfost et al., 2013; McGeown et al., 2015; Jerrim and Moss, 2019; Torppa et al., 2019) or whether a study explicitly asks for all sorts of different reading material in sum (e.g., Miyamoto et al., 2019). In some studies, there was no explanation for the participants regarding what reading material to in- or exclude when responding to a question on the reading time per week or the reading frequency (e.g., McElvany et al., 2008; Pfost et al., 2010; Schmidt and Retelsdorf, 2016), which means that these studies used an implicit summary of reading material.

### 5.4. Leisure to school

The example studies we discussed, especially those using global and differentiated evaluations, asked about reading for enjoyment (OECD, 2018), reading because one wants to OECD (2018), reading during leisure time (McGeown et al., 2015; Torppa et al., 2019) or outside of school (Miyamoto et al., 2019). At first glance, all of these studies seem to examine reading behavior in a similar reading context, namely the leisure context. However, upon closer look, there is some lack of clarity. Reading for enjoyment is not necessarily equivalent to reading during leisure time. First, reading for enjoyment does not necessarily take place during leisure time (e.g., reading for enjoyment in school). Conversely, leisure time reading does not necessarily mean that a person reads for enjoyment. Leisure time reading if not defined in more detail, as for instance done by Guthrie et al. (1999) does not exclude reading done for benefit-oriented purposes, such as reading school books/texts, for instance. Second, when a person is asked about time spent reading for enjoyment, this refers more to a specific purpose rather than a specific context. Therefore, research that asks participants about “reading for enjoyment” is measuring a construct that is very close to specific types of reading motivation.

## 6. Limitations

In terms of objective and structure, this article does not follow the classical literature review. Among other aspects, because it combines an analysis of existing research literature and conceptual ideas. This approach can be debated. Furthermore, the discussed studies represent a small selection that was made by the authors, to illustrate differences in applied measurement approaches as good as possible. We are aware that there is much more literature that could have been discussed within the scope of this article. In fact, there is an almost unmanageable number of articles dealing with *reading* and referring to l*arge-scale assessments* or *surveys*. A comprehensive, systematic literature review, e.g., with the aim of categorizing all measurement approaches with our proposed multidimensional distance measurement, also taking into account the results of the studies, could be a next step.

## 7. Conclusion

There is large variation in the measurement of the construct of reading behavior within large-scale assessments and surveys. By discussing the selected example studies, important, non-trivial differences within the applied measures between studies that aimed to address comparable research questions emerged. Often, researchers may not be fully aware of these differences. Hence, clarifying and addressing such differences in the applied measures might help to sharpen the focus. We think it is worth that future research takes such measurement differences into account while planning, conducting and interpreting research on reading behavior. The elaboration of the four dimensions and its categories hopefully might support such decisions and improves the comparison of results between existing studies.

In contrast to, for example, reading motivation, where valuable work has already been conducted to provide more clarity regarding the nature of the construct and thus research and its results (e.g., Schiefele et al., 2012; Conradi et al., 2014; Toste et al., 2020), conceptual studies on the reading behavior construct are scarce (e.g., Wimmer and Ferguson, 2022, for an exception). There are hardly any attempts by researchers in the field to examine the terminology of reading behavior in depth, either theoretically and conceptually or at the methodological and operationalization level. The present paper makes a first step, by discussing and organizing common operationalizations and measurement methods in large-scale assessments and surveys. However, this is just a starting point. Further discussion as well as theoretical and conceptual work is needed. There is a clear need for a theory of reading behavior in order to capture this construct with its subdimensions.

## Author contributions

FL: conceptual development of the article, literature research for the review, and leading author of the manuscript. MP: collaboration in the conceptual development of the article and the writing of the manuscript (e.g., feedback, improvements, and writing of smaller passages). Both authors contributed to the article and approved the submitted version.

## Conflict of interest

The authors declare that the research was conducted in the absence of any commercial or financial relationships that could be construed as a potential conflict of interest.

## Publisher’s note

All claims expressed in this article are solely those of the authors and do not necessarily represent those of their affiliated organizations, or those of the publisher, the editors and the reviewers. Any product that may be evaluated in this article, or claim that may be made by its manufacturer, is not guaranteed or endorsed by the publisher.

## Footnotes

^1^"How much time do you normally spend reading for enjoyment? Please take into account diverse kinds of reading, such as books, magazines, newspapers, websites, blogs, emails…."

^2^"How much time do you normally spend reading outside school on a normal school day/on weekend/holiday? Please consider all possible opportunities you have for reading, in other words not only books or magazines, but also e-mails or the Internet."
